# Acidic condition accelerates cation release from purple rock in Southwestern China

**DOI:** 10.1038/s41598-022-14851-1

**Published:** 2022-07-06

**Authors:** Jixia Zhao, Chunpei Li, Chuanhao Lu, Limei Deng, Gangcai Liu, Maopan Fan

**Affiliations:** 1 College of Resources and Environment, Yunnan Agricultural University, No. 95, Heijin Road, Panlong District, Kunming, 650201 China; 2 Key Laboratory of Mountain Surface Processes and Ecological Regulation, Chinese Academy of Sciences, Institute of Mountain Hazards and Environment, Chinese Academy of Science & Water Resources Ministry, No. 9, Block 4, Renmin South Road, P.O. Box: 417, Chengdu, 610041 China

**Keywords:** Geochemistry, Geomorphology

## Abstract

In spite of the fact that rock weathering performs an essential task in the evolution of the Earth’s surface, the quantitative assessment between pH and rates of chemical weathering remain unclear. This study aims to characterize the chemical weathering rate of purple rocks and then develops a model to calculate the release rates of cations (K^+^, Na^+^, Ca^2+^ and Mg^2+^) under various pH conditions. Two types of purple rock were sampled from the Shaximiao Group (J_2_s) and Penglaizhen Group (J_3_p), and a series of laboratory experiments were performed by soaking the purple rocks in solutions with pHs from 2.5 to 7.0, over 24 treatment cycles. The results showed that the release rates of cations apparently increased as the pH decreased. The release of Ca^2+^ was the dominant process of chemical weathering in J_3_p under various pH treatments, while K^+^ and Na^+^ were remarkably high in J_2_s (with the exception of the pH 2.5 treatment). Quantitative analysis revealed that the rate of cation release was significantly related to the H^+^ concentration (*p* < 0.001) and the air temperature (*p* < 0.001). The relationship between cation release and acidity was found to be an exponential function. Our results suggested that solution acidity serves as an important driving force for cation release rates from purple rocks and that environmental acidification would enhance rock weathering.

## Introduction

Weathering is one of the drivers of landform and soil development. The chemical weathering of rocks provides the cations that determine the long-term availability of plant nutrients^1,2^. Weathering changes drastically the physical properties of bedrock transforming the topography and influencing the relief evolution in different tectonic settings^2–4^. Additionally, the mineral weathering of bedrock is usually considered to be a crucial stage in the geochemical cycle because it directly affects all terrestrial life^5^. For example, if the balance between cation release via chemical weathering and loss through plant uptake is upset, then soil acidification occurs^6,7^. Therefore, the rate of cations that are released from parent materials through chemical weathering is a critical concern when investigating soil fertility, plant nutrient supply, buffering capacities of soils and surface water quality^8,9^.

The rate of chemical weathering is controlled by rock composition, climatic conditions (e.g., temperature and precipitation), topography, and biological activity^10–15^. Although a number of investigations have revealed the pH dependence of the slake-durability of rocks^16,17^, the influences of acids on the chemical weathering characteristics during purple rock disintegration have not been fully investigated. Recently, the combination of acid precipitation (known as ‘acid rain’) and secondary pollutants (formed through SO_2_, NO_x_ and NH_3_ emissions) has become of an increasing global environmental concern. China has become the third largest global source of acid deposition, following North America and Europe^18^. Further, acidic rain in both small mountain villages and great cities has been observed, especially in the regions to the south of the Yangtze River, in the basin of Sichuan, and to the east of the Qinghai-Tibet Plateau^19^. Acid deposition may accelerate leaching losses of important nutrient cations such as Ca^2+^ and Mg^2+^ and mobilise toxic Al^3+^ in the soil solution, which is detrimental to the fertility of soils^20,21^. Related studies have shown that acidic precipitation not only directly accelerates chemical weathering rates through the input of H^+^ ions^14^, but also indirectly drives cation release through the proton production from nitrogen transformation^22^. The rate of chemical weathering is therefore crucial to determining whether soils are sensitive to the effects of acid, which is usually quantified by measuring solute concentrations and flux^23,24^. Past research has focused on the effects of changing temperature and reactive fluid composition on the chemical rates of individual minerals in the laboratory^25,26^. However, rocks consist of many different minerals with distinct reactive and surface areas; thus, different have distinct responses to pH and temperature. Therefore, the rate at which cations are released through rock weathering under acidic precipitation has always been difficult to measure and quantify because of a variety of environmental factors.

**Figure 1:**
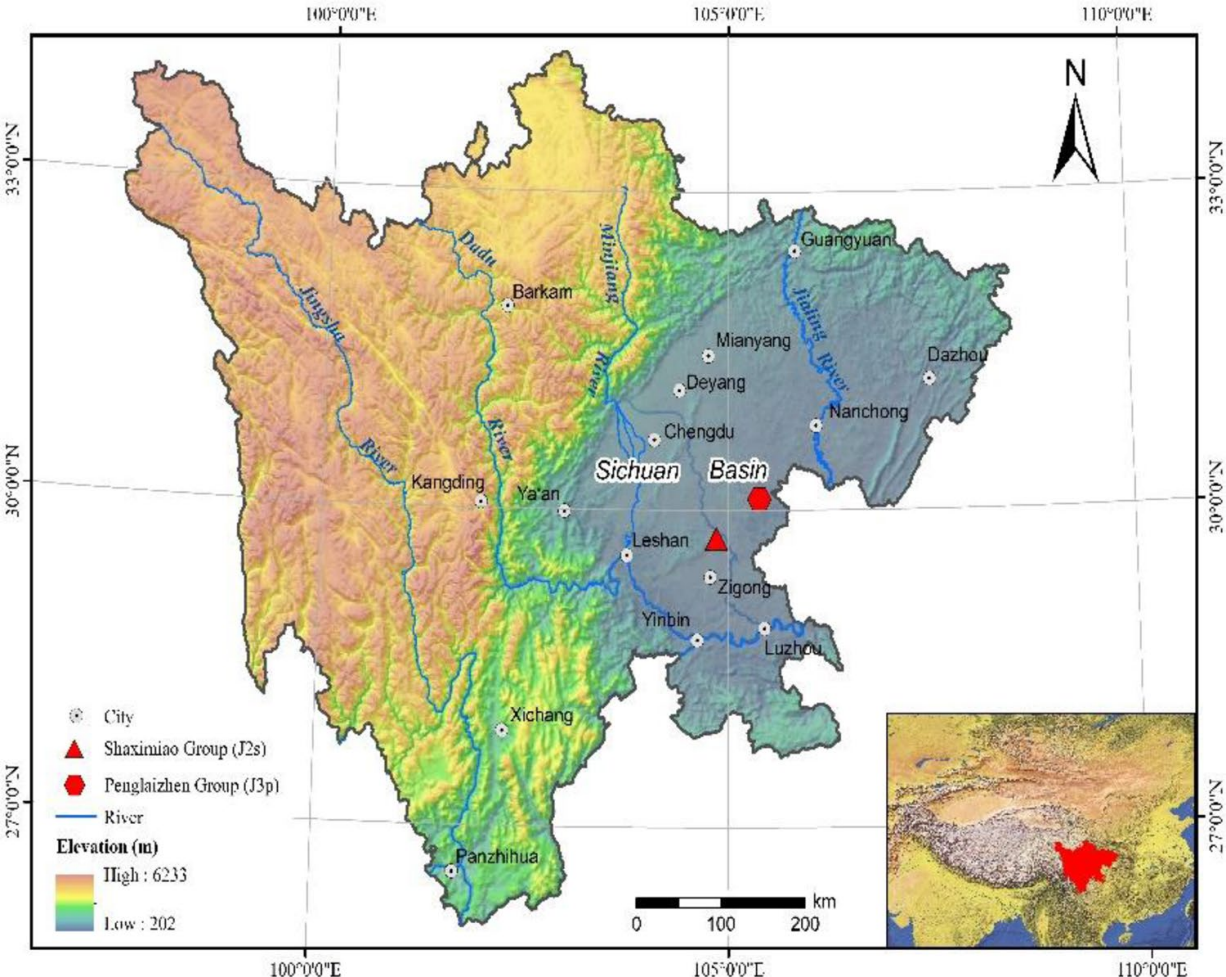
Location of the sampling sites. The map created by QGIS3.16, https://www.qgis.org/en/site/.

Purple soils (Regosols as classified by the Food and Agriculture Organization [FAO] Taxonomy or Entisols as classified by the United States Department of Agriculture [USDA])^27^ are formed from purple rocks (mudstones and/or sandstones) or their weathering products. These cover about 18 million ha in China and more than 75% of soil type in the upper Yangtze River, most commonly in the Sichuan basin of southwestern China by maintaining current topography of high in the northwest and low in the southeast, and where it is surrounded by the highlands of the Plateau of Tibet on the west and the Yunnan-Guizhou Plateau on the south and the Wu Mountains on the east and the Daba Mountains on the north, all of which protect the interior from temperature extremes (Fig. 1). The main characteristic of purple rocks is that they are easily decayed and very soft, and readily develops structural, diagenetic and decayed cracks. With rapid weathering, the purple rocks are easily broken up into rock fragments or gravel through anthropogenic activities, and crops can be planted directly in these. The physical weathering rate of purple rocks is much higher than other types of parent rocks under the same condition; He et al.^27^ found that the soil erosion modulus of purple rock was 15,800 t km^−2^ year^−1^, whereas it was only 55 t km^−2^ year^−1^ for limestone in the same area. Previous studies mainly focused on the effects of temperature and moisture on the disintegration characteristics of purple parent rock^28,29^. There’s little focus on the mineral release during the weathering process and the impact of acid solution, leading to it difficult to predict the effects of acid precipitation or environment acidification on the weathering rate of purple rock. The main distribution area of purple mudstone in southwestern China is a subtropical monsoon climate zone, and it is also sensitive to acid deposition. With the recent rapid development of industrial and agricultural activities, the problem of acidification is likely to worsen. Therefore, in this study, we aim to determine the quantitative effects of varying acidic solutions on rock decay when it is applied to purple rock by using controlled laboratory experiments.

## Materials and methods

### Materials

**Figure 2:**
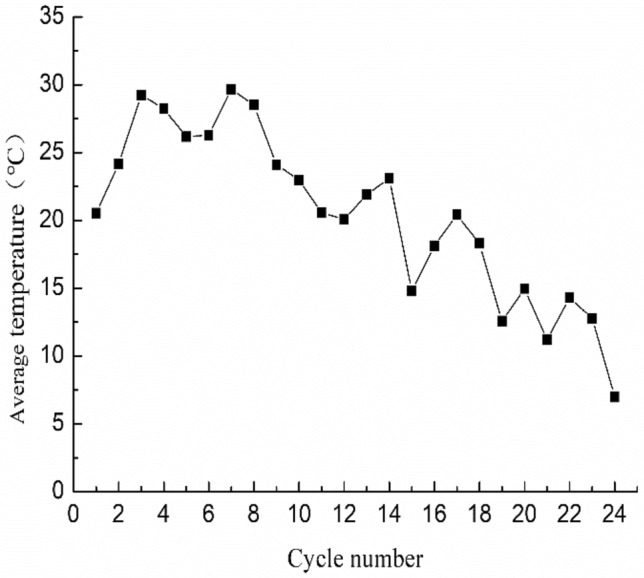
Average air temperature during each soaking treatment. The figure created by OriginPro 2021, http://www.Originlab.com/OriginProLearning.aspx (the same of Figs. 3, 4 and 5).

The samples of fresh purple rocks utilized in the experimental measurements were chosen from purple and red-brown mudstones of the group of the Shaximiao Group (J_2_s) (E104° 51′, N29° 44′), and the purple and brown sandy mudstone of the Penglaizhen Group (J_3_p) (E105° 23′, N30° 6′) in Sichuan Basin, all laid down in the Jurassic period, which accounts for 26.23% and 35.02% of the purple parent rock distribution area in Sichuan Basin. Sichuan Basin possesses a subtropical, monsoon climate with a yearly mean temperature between 14 and 19 °C and average yearly precipitation of around 850 mm. Most of the precipitation occurs between May and October. To better reflect the authenticity of weathering characteristics in an acidic environment, the fresh purple rock samples without acid effects were selected. Then, all of the samples were taken and treated at the Field Observation and Science Research National Station of Farmland Ecosystem in Yanting, Sichuan of China (N31° 16′, E105° 27′) where the temperature ranged from 7.0 and 29.7 °C and showed a fluctuating downward trend throughout the trial period (Fig. 2). The clay mineral content of the tested samples (Table 1) was determined using the X-ray diffraction method^30^. The elements K, Na, Ca and Mg (Table 2) were determined by atomic absorption spectrophotometry. Si, Al, Fe and Ti were using the gravimetric-molybdenum blue photometric method, the fluoride replacement method, the dichromate volumetric method, and the hydrogen peroxide photometric method, respectively^31^.

**Table 1:** Mineral contents (%) of the two purple mudstones.

Rock types	Montmoril-lonite	Illite	Kaolinite	Chlorite	Quartz	Potassium feldspar	Plagio-clase	Calcite	Dolomite
J_2_s	13	21	6	7	41	2	10	0	0
J_3_p	5	23	0	11	38	1	8	12	2

**Table 2:** Major chemical element contents (%) of the two purple mudstones.

Rock types	SiO_2_	Al_2_O_3_	Fe_2_O_3_	TiO_2_	K_2_O	Na_2_O	CaO	MgO	CaCO_3_
J_2_s	61.51	16.21	6.55	0.78	2.59	1.29	0.86	2.04	2.15
J_3_p	58.26	13.03	4.68	0.64	2.43	0.87	5.01	1.25	14.65

About 58% of precipitation in southwestern China has a pH value between 4.5 and 5.6^32^. The outcomes of acidic rain surveyed by the Sichuan Meteorological Bureau of Dazhou, Xichang, Chengdu, Panzhihua, E’meishan, and Anyue from 2006 to 2013 demonstrated that in Sichuan province, the average frequencies of stronger acids (pH ≤ 4.0) are about 17% and the average pH of precipitation is about 4.74^33^. Moreover, it has been detected that the pH value of precipitation in some regions of the Sichuan Basin was very low and ranged from 3.0 to 3.5^34,35^, and the nitric acid and sulfuric acid were the fundamental resources of acidity within the precipitation. The molar ratio of SO_4_^2−^ to NO_3_^−^ is around 4:1^36,37^. On the basis of this range, a mixture of synthetic acid solutions comprising H_2_SO_4_ and HNO_3_ with a ratio of the molar concentrations of around 4:1 was prepared. However, a pH lower than 3.0 is unusual in natural environments, the outcomes of acidity on chemical weathering are relatively long procedures, and in the simulation assessment, a lower pH is frequently utilized to simulate the long-lasting impacts of an acidic media within the natural environments for diminishing the involvement of humidity, temperature, and other external parameters^38^. Hence, the preparation of acidic solutions with values of pH of 5.6, 4.5, 3.5, and 2.5 was carried out through dilution of the stock solution by utilizing deionized water. In these experiments, the deionized water (with a pH of 7.0) was utilized as a control (CK).

### Experimental procedure

To ensure homogeneity, the samples for each treatment were selected from the same block of purple rock. Cut the parent rock sample into small pieces, and each cube was then shaped into a rough block with a mass of 660 ± 3 g. All of the tests were conducted in triplicate with the following detailed procedure.

All the prepared experimental samples were randomly placed into 500 ml glass beaker. Then, add 300 ml of acid solution to each beaker, and it was ensured that all the samples were submerged. After soaking for 72 h, each solution was filtered and collected to determine the ion (K^+^, Na^+^, Ca^2+^, Mg^2+^) concentration in solution. Put the residual rock cuttings on the filter paper back into the beaker and dried for more than 24 h at 105 °C to a constant weight. All of these decayed products were prepared for the next acid solution treatment after cooling, this procedure represented one treatment cycle. Particles of weathered products less than 2 mm in diameter are commonly regarded as soil particles^27,39^. Therefore, a total of 24 cycles were carried out for all of the treatments because the disintegration rate of the purple rock treated with each acid solution were exceed 90%, which indicated that the disintegration process has basically stopped. Herein, the pH of the collected filtered solutions was determined by a pHS-3E acidimeter, and ion chromatography was used to determine Na^+^, K^+^, Ca^2+^ and Mg^2+^ in the filtered solution.

### Calculation of cation release in the treatment cycle

Based on the solution volume and the ions collected in each soaking test, the amount of K^+^, Na^+^, Ca^2+^ and Mg^2+^ in the filtered solution were calculated in accordance with the following Eq. (1):

1 $${S}_{i}={C}_{i}\cdot {V}_{i}/({M}_{i}\cdot m\cdot 1000),$$

where ${S}_{i}$ represents the released amounts of K^+^, Na^+^, Ca^2+^ and Mg^2+^ during each treatment cycle (mmol kg^−1^); $i$ is K^+^, Na^+^, Ca^2+^ and Mg^2+^; ${C}_{i}$ is the concentration of each cation in the filtered solution (ppm); ${V}_{i}$ represents the volume of filtered solution collected after each soaking (ml); ${M}_{i}$ is the relative atomic mass of each atom (K-39, Na-23, Ca-40 and Mg-24) and $m$ indicates the weight of the soaked rock (kg).

As a result, the release rate of cations is expressed by Eq. (2):

2 $${V}_{T}=\sum {S}_{i(i={K}^{+},{ Na}^{+}, {Ca}^{2+},{Mg}^{2+})}/t,$$

where ${V}_{T}$ is the total release rate of the cations K^+^, Na^+^, Ca^2+^ and Mg^2+^ in each treatment cycle (mmol kg^−1^ h^−1^), which was used as an index to measure the weathering rate^40^ and $t$ is the duration time of each soaking (h).

### Data analysis and statistics

Multiple comparisons are used to analyze the effects of different treatments and parent rock types on the amounts of cation release. Analyses of the least significant difference (LSD) tests were performed using the SPSS v.12.0 software to determine whether the amounts of cations released were significantly different for the various acid treatments. After regression analysis, the models of the relationship between the variables were set up via the regressive determination parameters compared by the OriginPro 2021 software. The statistically significant level was set at *p* < 0.05, and the statistical parameters for all of the data were then analyzed using SPSS v.12.0.

To effectively describe the reaction of rock to acidic experimental environments in the laboratory, a total of 120 sets (24 cycles of five treatments) of data were analyzed, including chemical weathering rates, H^+^ concentration of the soaking solution and natural temperature. Using nonlinear, multivariate regression, Eqs. (3) and (4) were formulated:

3 $$V_{T} = e^{{ac}} (H^{ + } ) \cdot {\text{ }}\left( {\frac{A}{{RT}}} \right)^{\beta } ,$$

where ${V}_{T}$ is the same as in Eq. (2), ${c}_{({H}^{+})}$ is the concentration of H^+^ (mmol L^−1^), *T* is temperature (K) (Fig. 2), *R* is the gas constant (kJ mol^−1^ K^−1^) and $a$, $\beta$ and $A$ are constants to be determined by regression analysis and are dependent on rock material properties.

4 $$\mathrm{y}=k{e}^{(-\mathrm{x}/\gamma ))}+\delta ,$$

where y is the released amount of cations, x is the pH of soaking solution, *k*, $\gamma$, $\delta$ are constants to be determined by regression analysis and are dependent on rock and were able to correctly.

## Result

### Release of cations in the soaking solution

**Figure 3:**
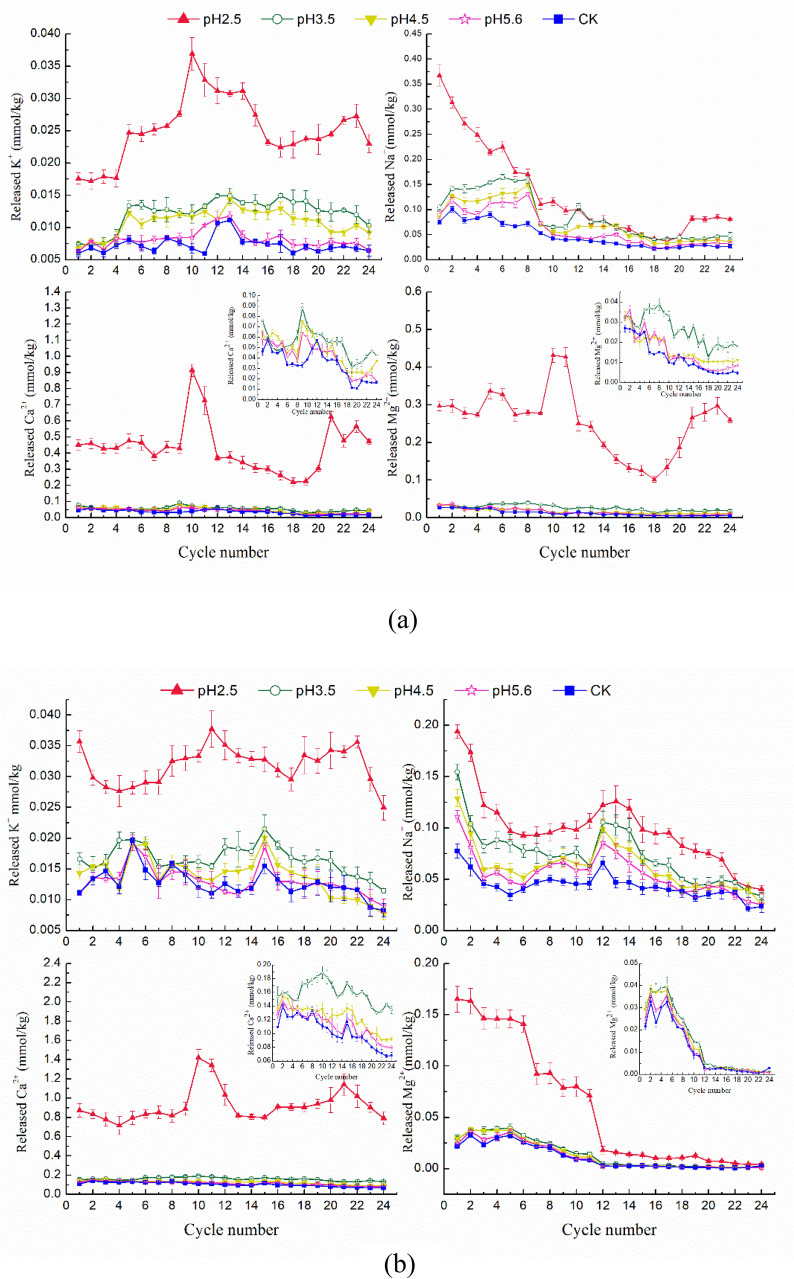
Cations released in the soaking solution from J_2_s (**a**) and J_3_p (**b**) after each treatment cycle. The inserts in figure show enlargements of the parts of released Ca^2+^ and Mg^2+^ with the pHs of 3.5, 4.5, 5.6 and CK.

The variation trend of cations in the soaking solution of parent rocks under different acid treatments is similar, that was, the stronger the acid treatment accompanied with the higher the release amount of each cation (Fig. 3). During the whole soaking process, the release amount of each cation under pH 2.5 treatment was significantly greater than that under other acid treatments. The K^+^ release process of the two groups of parent rocks under pH 2.5 immersion showed a single peak curve, and obvious peaks appeared in the middle stage of immersion, with the release amounts of 0.037 mmol kg^−1^ and 0.038 mmol kg^−1^ from J_2_s and J_3_p respectively, and then showed a fluctuating downward trend to tend to be stable. The released K^+^ of J_2_s under pH 3.5, pH 4.5, pH 5.6 and CK treatments were relatively gentle, with little fluctuation and unobvious peak value. However, the K^+^ release from J_3_p showed two obvious peaks during soaking between pH 3.5 and pH 7.0, and the release showed a slow downward trend after the second peak change. The release laws of Na^+^ between two parent rocks were basically similar, that the overall trend was fluctuating and declining with the soaking process. Na^+^ release of J_3_p under different pH acid solution treatment had obvious fluctuation peak in the middle stage of immersion, while it decreased continuously from the beginning of soaking in the J_2_s. Na^+^ increased slightly under pH 2.5 treatment in the later stage of soaking, while under other pH treatment Na^+^ release decreased slowly and the curve changed gently. The Ca^2+^ release curves of J_2_s and J_3_p in strong acid (pH = 2.5) solution were similar, which had a single peak curve trend, and arrived the peak value of 0.912 mmol kg^−1^ and 1.417 mmol kg^−1^ respectively at the 10th immersion. The Ca^2+^ release laws of J_2_s under pH 3.5 to pH 7.0 were similar to that under the treatment of strong acid solution with pH 2.5, and a relative high peak appears in the middle stage of soaking, and then had a fluctuating downward trend. However, the Ca^2+^ release curves of J_3_p under the treatment of acid solution with pH value of 3.5–7.0 were consistent, and the Ca^2+^ release amount were relatively high at the beginning of soaking. With the soaking cycle, the Ca^2+^ release curve fluctuated and the decline range was large at the beginning stage, and it decreased slowly or tends to be gentle at the later stage. The release law of Mg^2+^ under different acid treatments were similar to that of Na^+^. With the progress of soaking process, the overall trend of Mg^2+^ were fluctuating and declining, and the release of Mg^2+^ rapidly decreases to a relatively low value during the middle stage of soaking. From the overall trend, it is easy to find that leaching of the cations from the purple rock were affected most strongly by pH. With the pH of soaking solution decreases, the H^+^ input increased as well, which not only provide more protons to exchange with the active and adsorbed cations, but also promote minerals weathering. Add to all that its results caused more cations release and leaching from minerals.

### The effect of pH on the amount of cations released

The amounts of elements released by an acid attack in laboratory experiment (the unit charge of elements released from 1 kg of soil during the weathering process) were utilized as a criterion to assess the rate of weathering^40^. In this study, the amounts of K^+^, Na^+^, Ca^2+^ and Mg^2+^ released from J_3_p and J_2_s under the effects acid solutions were obvious after 24 treatment cycles. As Table 3 showed, the acid solutions caused more cations to be released than the deionized water for the two rock types. The whole amount of cations released from all of the studied types of rocks enhanced as pH diminished, particularly when pH was less than 3.5, and a significant difference (*p* < 0.05) was observed after the acid treatments. Compared with the control treatment of pH 7.0 (CK), the amount of cations released from sample J_3_p after treatment with solutions of pH 2.5, 3.5, 4.5 and 5.6 increased by multiples of 6.50, 1.55, 1.25 and 1.14 times, respectively. Comparable values were 8.23, 1.74, 1.41 and 1.22 times for J_2_s. Compared to the CK, the Ca^2+^ released from the pH 2.5 solution apparently increased by 19.59 and 9.59 mmol kg^−1^ for J_3_p and J_2_s, respectively, and Mg^2+^ increased by 1.20 and 5.82 mmol kg^−1^. Ca^2+^ was the predominant cation released under the various acid solutions, with the average Ca^2+^ amount accounting for 49.80% for the two rock types, while Na^+^ represented 33.14%, Mg^2+^ 10.96% and K^+^ 6.09%.

**Table 3:** Amounts of cations released from purple rock under various pH weathering conditions.

Rock types	pH	Amount of cations released (mmol kg^-1^)			
		K^+^	Na^+^	Ca^2+^	Mg^2+^
J_2_s	2.5	0.61 ± 0.03a	3.19 ± 0.21a	10.43 ± 0.20a	6.12 ± 0.05a
	3.5	0.29 ± 0.02b	2.09 ± 0.13b	1.30 ± 0.03b	0.62 ± 0.03b
	4.5	0.26 ± 0.01c	1.78 ± 0.14c	1.05 ± 0.05c	0.39 ± 0.02c
	5.6	0.20 ± 0.01d	1.49 ± 0.14d	0.97 ± 0.03c	0.36 ± 0.01c
	CK	0.18 ± 0.01de	1.15 ± 0.07e	0.84 ± 0.02d	0.30 ± 0.02d
J_3_p	2.5	0.76 ± 0.02a	2.37 ± 0.28a	22.08 ± 0.25a	1.46 ± 0.05a
	3.5	0.40 ± 0.01b	1.77 ± 0.17b	3.82 ± 0.05b	0.35 ± 0.01b
	4.5	0.33 ± 0.01c	1.49 ± 0.14bc	2.97 ± 0.02c	0.32 ± 0.01bc
	5.6	0.32 ± 0.04c	1.33 ± 0.15cd	2.72 ± 0.03d	0.29 ± 0.01cd
	CK	0.30 ± 0.03c	1.05 ± 0.11e	2.49 ± 0.05e	0.26 ± 0.01e

Different letters in a column indicate significant differences between the treatments, where *P* < 0.05.

### Relationship between the amount of cations released and pH

**Figure 4:**
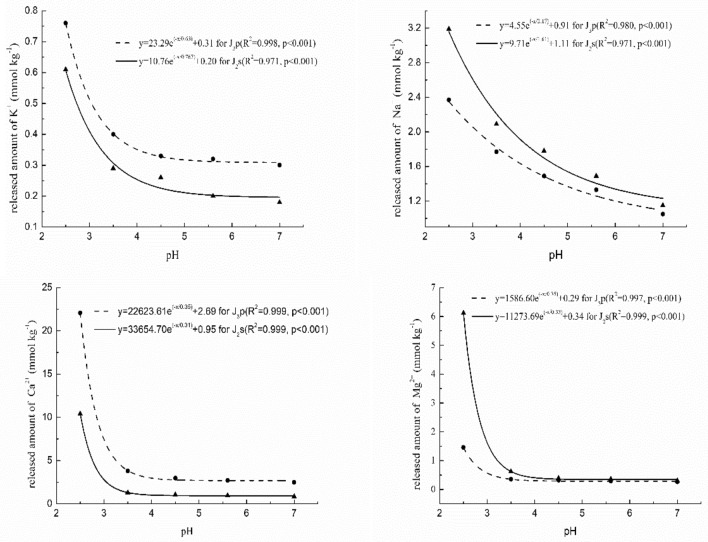
Relationship between the total cations (K^+^, Na^+^, Ca^2+^ and Mg^2+^) released and pH.

As expected, the stronger the acid treatment, the higher the amount of K^+^, Na^+^, Ca^2+^, and Mg^2+^ in the leachates of the two rock groups. Regression analysis showed that the amount of released individual cations appeared to exponentially decrease as pH increased in all two of the rock types tested. The exponential correlations between pH and cation release were significant (*p* < 0.001), especially for the amount of Ca^2+^ and Mg^2+^ released, with correlation coefficients (*R*^2^) above 0.999 (Fig. 4). This also illustrated that the sensitivity of cation release to pH variation for the various cation types was high for all two rock types and that Ca^2+^ and Mg^2+^ were more apparent than K^+^ and Na^+^. On the whole, these data fit the exponential Eq. (4) and quantitatively predict the cation amounts released from purple rock under varying acidic conditions in the laboratory.

### Determination of the quantitative relationship between cation release rates and H^+^ concentrations

Intuitively, it can be expected that acid would exert a strong influence on chemical weathering; however, this effect may vary with environmental changes such as air temperature. In general, the net total output of cations (K^+^, Na^+^, Ca^2+^ and Mg^2+^) is used to represent ‘chemical weathering’^41^; therefore, to provide credible estimates, the chemical weathering rate in acidic environments should be considered and incorporated into future models. In this study, the chemical weathering rate of cations was calculated and combined with other variables in Eq. (3) using SPSS v.12.0, and the results showed that the data fit the model to a significant degree (*p* < 0.001). The correlation coefficients of the cation release rates related to the concentrations of H^+^ were 0.897 and 0.767 for J_3_p and J_2_s, respectively, and they were apparently greater than those related to temperature, which were 0.045 and 0.116 for J_3_p and J_2_s, respectively. The multiple correlation coefficients (${R}^{2}$) were above 0.883 and were higher than the partial correlation coefficients (Table 4). Thus, our results showed that the model of chemical weathering rates was not satisfactory when the rate was determined only based on solution acidity (pH) without consideration of the temperature effects.

**Table 4:** Quantitative model of cation release rates for the two groups of rock.

Rock groups	Regression equations	Correlation coefficients (${R}^{2}$)	Partial correlation coefficients (${R}_{{c}_{({H}^{+})}}^{2}$,${R}_{T}^{2}$)		*p*
J_2_s	${v}_{T}={e}^{0.61\cdot {c}_{({H}^{+})}}\cdot {(\frac{3853.10}{RT})}^{-14.02}$	0.883	0.767	0.116	< 0.001
J_3_p	${v}_{T}={e}^{0.56\cdot {c}_{({H}^{+})}}\cdot {(\frac{5472.96}{RT})}^{-7.34}$	0.942	0.897	0.045	< 0.001

## Discussion

A remarkable number of laboratory investigations have illustrated the essential role of pH in the processes of chemical weathering^42,43^. In this study, our results indicates that the concentration of H^+^ in the reaction environment is an important impact factor for weathering kinetics, consistent with those of previous studies^14,44–46^. The rate of the release of cations from the purple rocks obviously enhanced as the solution pH diminished, especially when the value of pH was less than 3.5 (Table 3). Similarly, the effect of acidification on chemical weathering is low^47^, therefore, long-term evaluation is necessary to reveal the effects of acid deposition on chemical weathering^46^. In the current research, the performed analysis of cation release divulged substantial impacts of pH on chemical weathering among the two purple rocks, particularly when the amount of Mg^2+^ and Ca^2+^ released during the pH 2.5 processing was considered. It indicated that with the increasing of the acidity of the solution promoted the amount of leaching of divalent cations, but has less influence on the leaching of univalent cations, which suggests that the differences of univalent cations leached from purple rock between the acid- and water-eluted became small. These effects were attributed to the fact that compared to univalent cations, the divalent cations released were more affected by the pH of the environment. The effects were significantly greater than those caused by the other treatments, which was attributed to the fact that compared to univalent cations, the divalent cations released were more affected by the pH of the environment^48,49^. Calcium in the rock mainly exists in inorganic form, and inorganic calcium can be further divided into mineral and exchange state calcium^50^. Of these, mineral calcium mainly exists in the crystal lattice of the rock in its solid phase and can seldom be released through hydrolysis. In contrast, the calcium-bearing minerals in purple rock are mainly silicate minerals and carbonate minerals. The acidities of silicic acid and carbonic acid are weaker than those of the simulated solutions due to the action of mixed acids of SO_4_^2−^ and NO_3_^−^. In this way, the calcium within silicate and carbonate minerals can be easily dissolved by acid. In contrast, the released quantity of potassium is evidently lower than that of calcium, which is mainly because most of the calcium-bearing primary minerals in the rock are easily weathered. In particular, the weathering rate of calcium-bearing plagioclase is much higher than that of potassium feldspar or sodic feldspar^50^. Meanwhile, the lattice energy of calcium-bearing minerals is much smaller than those of sodium- or potassium-containing minerals of the same type^50^. Additionally, the major clay minerals of the purple block (including illite, montmorillonite, and chlorite) are all phyllosilicate-based minerals with a ratio of 1:2 in illite and montmorillonite and in chlorite a ratio of 2:1:1^51^. The reactions of these minerals in acidic solutions may represented as follows:

Montmorillonite^52^:

$$\begin{aligned} & ({\text{K}}_{{0.0{2}}} {\text{Na}}_{{0.0{5}}} {\text{Ca}}_{{0.{41}}} {\text{Mg}}_{{0.{18}}} )({\text{Mg}}_{{{1}.{11}}} {\text{Fe}}_{{0.{17}}} {\text{Al}}_{{{2}.{77}}} )({\text{Al}}_{{0.{3}}} {\text{Si}}_{{{7}.{7}}} ){\text{O}}_{{{2}0}} \left( {{\text{OH}}} \right)_{{4}} + {13}.{\text{2H}}^{ + } + {6}.{\text{8H}}_{{2}} {\text{O}} \\ & \quad \Rightarrow 0.0{\text{2K}}^{ + } + 0.0{\text{5Na}}^{ + } + 0.{\text{41Ca}}^{{{2} + }} + {1}.{\text{29Mg}}^{{{2} + }} + 0.{\text{17Fe}}^{{{3} + }} + {3}.0{\text{7Al}}^{{{3} + }} + {7}.{\text{7H}}_{{4}} {\text{SiO}}_{{4}} . \\ \end{aligned}$$

Illite^53^:

$$\begin{aligned} & \left( {{\text{Si}}_{{{3}.{55}}} {\text{Al}}_{{0.{45}}} } \right)\left( {{\text{Al}}_{{{1}.{27}}} {\text{Fe}}^{{({\text{III}})}}_{{0.{36}}} {\text{Mg}}_{{0.{44}}} } \right){\text{O}}_{{{1}0}} \left( {{\text{OH}}} \right)_{{2}} \left( {{\text{Ca}}_{{0.0{1}}} {\text{Na}}_{{0.{13}}} {\text{K}}_{{0.{53}}} } \right) + {7}.{8}0{\text{H}}^{ + } \\ & \quad \Rightarrow {3}.{\text{55SiO}}_{{2}} + {1}.{\text{72Al}}^{{{3} + }} + \, 0.{\text{36 Fe}}^{{{3} + }} + 0.{\text{44 Mg}}^{{{2} + }} + 0.0{\text{1 Ca}}^{{{2} + }} + 0.{\text{13 Na}}^{ + } + 0.{\text{53K}}^{ + } + {4}.{9}0{\text{H}}_{{2}} {\text{O}}{.} \\ \end{aligned}$$

Chlorite^54^:

$$\left( {{\text{Mg}},{\text{ Fe}},{\text{ Al}}} \right)_{{6}} \left[ {{\text{AlSi}}_{{3}} {\text{O}}_{{{1}0}} } \right]\left( {{\text{OH}}} \right)_{{8}} + {\text{16H}}^{ + } \Rightarrow \left[ {{6}\left( {{\text{Mg}},{\text{ Fe}},{\text{Al}}} \right)} \right]^{{{13} + }} + {\text{ Al}}^{{{3} + }} + {\text{ 3H}}_{{4}} {\text{SiO}}_{{4}} + {\text{6H}}_{{2}} {\text{O}}{.}$$

These reactions promoted the replacement of intercrystalline Ca^2+^, Mg^2+^, Fe^3+^ and Na^+^ by H^+^ in the solution. However, the crystal structure of illite, which accounts for the vast majority of the clay mineral composition of the rock, constitutes two tetrahedral sheets with face-to-face top oxygen molecules. Moreover, the layers are tightly stacked in a staggered arrangement, and a coordinate, co-edge, octahedral combination is produced by the superposition, forming an octahedral sheet (O). Thus, a basic structural layer is formed, the interlayer space of which is filled with K^+^ with an equilibrium electrovalence. The K^+^ exposed between the layers is directly absorbed onto the silica tetrahedra and is not easily replaced by H^+^ in the solution.

**Figure 5:**
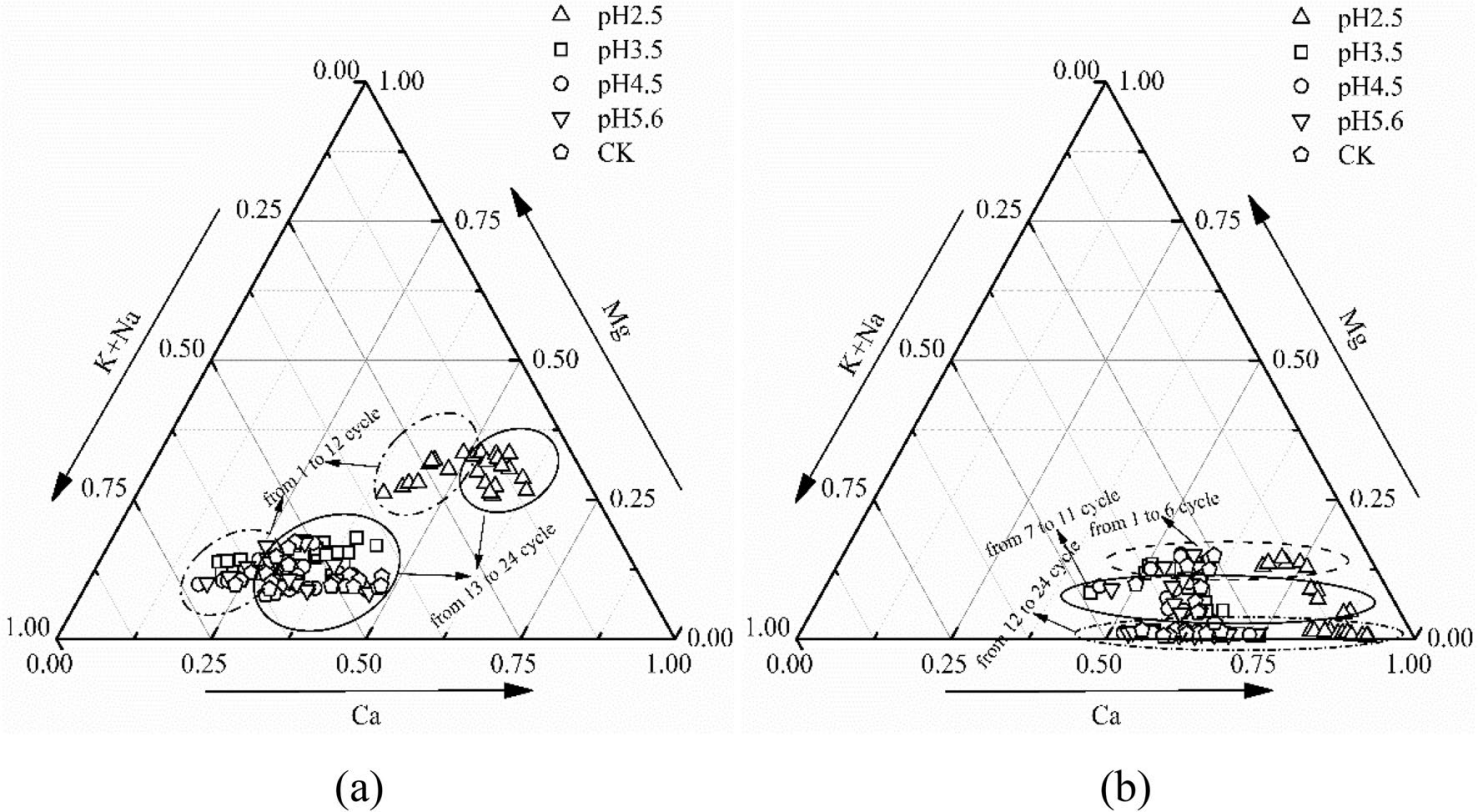
Ternary diagrams showing the cation compositions of purple rocks J_2_s (**a**) and J_3_p (**b**) from the filtered solutions used in the present study.

On the basis of transition state theory, chemical weathering processes may be influenced by the proton or aqueous activities of elements^55^. The distribution of cations (K^+^, Na^+^, Ca^2+^ and Mg^2+^) on the ternary diagrams presented in Fig. 5 can be used to indicate the relative contributions of major ions under various acidic environments^56^. Silicate- and carbonate-rich minerals are major components of the purple rock lithologies; therefore, considering that they can be weathered easily (especially carbonates), they are expected to contribute significantly to the major cation budget of the chemical weathering process^55^. Therefore, Ca^2+^ was the dominant species in J_3_p under various acid treatments, while K^+^ and Na^+^ were remarkably high in J_2_s except for the pH 2.5 treatments, because of the difference in mineral composition of the two purple rocks, In particular calcite in J_3_p is significantly higher than that in J_2S_. Notably, the relative contributions of major cations to chemical weathering varied with pH and time. The proportion of Ca^2+^ released by pH ≥ 3.5 treatments was substantially decreased when compared with the Ca^2+^ released by the treatment at pH 2.5. The proportions of Na^+^ and K^+^ behaved in the opposite manner, and the contribution rate of Mg^2+^ changed only slightly with changing pH. On average, the release of Mg^2+^ only accounted for a small fraction of the major cations, and its contribution in J_3_p approached 0 with an increase in time; it should also be noted that the contributions of Na^+^ and K^+^ increased.

Many researchers^57–59^ have studied the effects of pH and temperature on mineral dissolution kinetics. Previous studies indicated that the rate of water exchange around an octahedral cation can be correlated to the mineral dissolution rates in orthosilicates^60^. Regression analysis of our results revealed that the relationships between cation release rate, H^+^ concentration and natural temperature were significant for the two purple rock samples (p < 0.001). According to partial correlation coefficients (Table 4), both the H^+^ concentration and temperature significantly influenced the cation release rates (p < 0.001). That were related to the constituents of the parent rock materials, which include a considerable percentage of clay minerals, namely, illite, montmorillonite, chlorite, and previous studies have suggested that the clay mineral dissolution rates increase with temperature strongly depends on pH^61,62^ In addition, more studies have shown that the more quickly dissolution could be achieved of clay minerals by a departure from neutral pH circumstances, and at greater temperatures, the rate of solution is greater^63,64^. A thorough study on phyllosilicate weathering indicates that the rate of weathering is lesser at low temperatures and moderately acidic to neutral pH, and it is greater at higher temperatures and lower pH, which are divided through a transition zone^65^. Obviously, the rate of cation release was significantly related to the pH and the air temperature. Therefore, any attempts to model cation release rates based on the independent effect of pH alone and without consideration of temperature effects will be unsatisfactory, and our developed model (Eq. 3) would be more accurate for the prediction of cation release rates under environments with variable acidity.

## Conclusion

Our results show that the total amount of cations (K^+^, Na^+^, Ca^2+^, and Mg^2+^) released from purple rock during the chemical weathering process significantly (*P* < 0.001) increases with an increase in H^+^ concentration. The release of Ca^2+^ is the main contribution to cation release from the J_3_p under various acid treatments, while K^+^ and Na^+^ are the main cations released from the J_2_s, with the exception of the pH 2.5 treatment. The models for cation release rates fit the observed data well when the two variables are introduced (i.e., H^+^ concentration and air temperature), and there is a significant (*P* < 0.01) exponential relationship between the amount of cations released and the pH. Therefore, these models can be considered to be a baseline that will allow the cation release rates of purple rock to be quantitatively predicted under varying environmental acidification conditions. In conclusion, our results suggest that acidic condition will accelerate the chemical weathering of purple rock.

## Supplementary Information

Supplementary Information 1.

Supplementary Information 2.

Supplementary Information 3.

Supplementary Information 4.

## Data Availability

All the data generated and provided in the study is included under the main article and supplementary material file.
