# Supplementary material for: Acidic condition accelerates cation release from purple rock in Southwestern China
Source: Sci Rep. 2022 Jul 6;12:11412. doi: 10.1038/s41598-022-14851-1 (PMC9259688; doi:10.1038/s41598-022-14851-1)
Supplement: Supplementary file 2 — Supplementary Information 2. [file 41598_2022_14851_MOESM2_ESM.docx]

**Datas legends**

1. The file of “J2s.xlsx” including the date of K^+^, Na^+^, Ca^2+^, Mg^2+^ released in the soaking solution from Shaximiao Group (J_2_s) after each treatment cycle, and it’s the source data of Table3, Table4, Fig. 1 (a), Fig.2 and Fig. 4.
2. The file of “J3p.xlsx” including the date of K^+^, Na^+^, Ca^2+^, Mg^2+^ released in the soaking solution from Penglaizhen Group (J_3p_) after each treatment cycle, and it’s the source data of Table3, Table4, Fig. 1 (b), Fig.2 and Fig. 4.
3. The file of “Average air temperature.xlsx” including the date of average air temperature during each soaking treatment, and it’s the source data of fig.3
